# Editorial: Addressing epistemic injustice in mental health

**DOI:** 10.3389/fpsyt.2024.1382528

**Published:** 2024-03-12

**Authors:** Karen Newbigging, Anthony Salla, Ulla-Karin Schön, Colin King

**Affiliations:** ^1^ Independent Researcher, Malvern, United Kingdom; ^2^ Psychiatry, Oxford University, University of Birmingham and Centre for Mental Health, Oxford, United Kingdom; ^3^ City University of London, London, United Kingdom; ^4^ Stockholm University, Stockholm, Sweden; ^5^ Manchester Metropolitan University, Manchester, United Kingdom

**Keywords:** epistemic injustice and mental health, epistemic injustice and psychiatry, sanism, equalities, race and mental health

The relationship between knowledge and power is well established and in mental health the limitations of psychiatric knowledge well- rehearsed. In this context, disability and survivor movements have long been concerned with inequalities in knowledge production, and with action to address these and support empowerment.^1^ Epistemic injustice was conceptualised by Fricker as a form of social injustice, which occurs when people’s authority ‘as a knower’ is ignored, dismissed, or marginalized.^2^ The idea is attracting increasing interest in the mental health field because of the recognition of the asymmetries of power between people using mental health services and mental health professionals. People experiencing mental distress are particularly vulnerable to forms of epistemic injustice arising from deeply embedded social stigma, negative stereotyping, and assumed irrationality, amplified by other forms of systemic inequalities, notably race, gender sexuality, disability, and age.

The goal of this Research Topic is to examine how epistemic injustices in the mental health field occur and how epistemic justice can be advanced. It is essentially concerned with the question of whose knowledge counts and how can we ensure that lived experience is foundational to our knowledge about mental health. The fifteen papers in this Research Topic are wide-ranging and cover both theoretical and practical aspects of Fricker’s differentiated, but overlapping, aspects of epistemic injustice: i.e. testimonial injustice (the down grading and dismissal of individual testimony) and hermeneutical injustice (the absence or disadvantaging of collective interpretations and meaning of lived experience).

The first two articles by Russo and Levin, provide a critical examination of the concept of epistemic injustice and problematize its uncritical incorporation into the fields of psychiatry and mental health. Together they provide an invaluable reference point for the subsequent contributions. Russo raises concerns about the intellectualization of the idea of epistemic injustice and how it’s co-option by the psy-complex can exacerbate the marginalisation of lived experience, by not critically examining its foundations. She highlights how Mad Studies has the potential to address this by advancing first person knowledge, independent of the psy-complex; concluding with the hope that it further fosters hermeneutical justice. Foucault famously argued that knowledge is never neutral and reflects the operation of social power, providing a theoretical basis for the idea of epistemic injustice.^3^ In her perspective, Levin uses Critical Race Theory to consider alternative approaches to Foucauldian ideas about knowledge and power that challenge the presumed “superiority of “white, Western and modern ways of knowing the world”. In a similar vein to Russo, Levin argues for lived experience and the diversification of “knowledge about knowledge”.

Hultman and Hultman, a young disabled woman and her mother, use critical personal narratives to explore their lived experience of epistemic injustices in the Swedish mental health system. Their account brings to life the injustices described by the previous authors. Notably, the professionals’ willingness to tell the young woman what was wrong with her or to disbelieve her account of suicidal feelings. They describe a stark paradox that while the daughter’s disability was focused on, there was failure to provide support for her basic needs associated with this. Similarly, Bergen et al. focus on communication practices for people seeking emergency care for self-harm and suicidal ideation and self-harm in emergency departments in England. Using conversation analysis of video recordings of biopsychosocial assessments, their findings show how practitioners undermined service users’ lived experience through a variety of means including implying inconsistency and implausibility. They highlight how this can leave service users feeling more distressed and discouraged from help-seeking whilst acceptance and validation of experience leads to more positive outcomes. How potential service users are viewed in policy also shapes the service response and the support they may access. This is illustrated by Levin et al.’s policy analysis of discourses guiding provision for girls identified as being in distress and needing support from Israeli public social services. Their research shows how policies can play a critical role in “maintaining, shaping or correcting epistemic injustices.” They describe how the policy descriptions of girls in distress renders them as passive and voiceless and ignores the social context of their lives – conceptualizing this as existential epistemic injustice.

The legitimation of lived experience is, therefore, a critical axis for understanding and promoting epistemic injustice. This is the focus for Grim et al.’s study, which identifies practical barriers and facilitators to legitimation. They identify the need for shifting culture to integrate service user knowledge and propose a model to increase equality and the meaningful and sustainable co production of knowledge. This requires shifts in the current paradigm involving organisational and financial commitment. Nouf and Ineland contribute significantly to this academic discourse through a meta-analysis incorporating 544 narratives of lived experiences within mental health services in the Nordic countries. Their innovative contribution introduces the concept of “epistemic citizenship,” synthesizing the policy concept of ‘active citizenship’ with the theoretical construct of ‘epistemic injustice.’ Their findings shed light on the structural impediments that impede the establishment of arenas wherein service users are accorded the status of equal epistemic citizens.

The contributions from Hultman and Hultman, Grim et al., and Nouf and Ireland underline the central role that research methods play in knowledge construction through the delineation of the research question and the methods used. Okoroji et al. describe the experience of two third-sector organisations, in England, to explicitly address how power symmetries can be addressed in research. They highlight the problems of ‘elite capture’, such that participatory research can lack representativeness, and ‘epistemic exploitation’, such that “lived experience becomes a perpetual testimony with little influence”. The authors, therefore, advocate for a pragmatic approach that focuses on achievable change.

With the aim of informing the current Mental Health Act reform in England, Mooney et al. present a participatory model of research practice, using photovoice. Their contribution illustrates transformative research practices capable of acknowledging and valorizing lived experiences while concurrently addressing structural disparities, through accentuating the expertise of participants from racialized communities with experience of compulsory detention. As Crenshaw^4^ has powerfully argued systems of oppressions intersect to shape experience and amplify discrimination. Two further papers consider the intersection of race and mental health and propose action to address associated forms of epistemic injustice. Smith et al. detail the Patient and Carer Race Equality Framework, (PCREF). This framework aims to identify and redress racial disparities pervasive in mental health care in England and Wales. The authors underscore the guiding principles and priorities of the PCREF, elucidating its potential to rectify epistemic imbalances for individuals from racialized communities. One of the key aspects of the PCREF is the provision of culturally appropriate independent mental health advocacy (IMHA) to ensure that people from racialised communities are central to decisions about their care and treatment. Salla et al. explore the pivotal role of culture, race, and racism in IMHA provision, through the conceptual lens of epistemic injustice. They argue that it offers a mechanism to challenge prevailing racialised epistemic injustices and offer a conceptual framework for culturally appropriate advocacy, with learning domains at both individual and organizational levels for its potential to be realized.

Whilst the majority of papers have focused on service user experiences of epistemic injustice, Moberg and Schön, use it as a lens to explore how staff might support adolescents as epistemic subjects in the implementation of a patient-initiated brief admission in Sweden. They found that top-down decision making to implement the initiative and their minimal involvement in decision-making limited the epistemic agency of staff. They argue that the reduced agency of staff has implications for the sustainability of this initiative designed to promote the agency of young people in defining their support needs.

Finally, three papers focus on Child Sexual Abuse and the Independent Inquiry into Child Sexual Abuse (IICSA), which investigated whether public bodies and non-state institutions have taken seriously their responsibility to prevent and better protect children from sexual abuse in England and Wales. Historical Institutional Abuse Inquiries have increased over the last three decades bringing opportunities for survivor and victim participation. Despite this, a knowledge gap has existed in understanding the implications of this participation, and learning from research approaches which can challenge epistemic injustice of CSA. Barker et al. elucidate how engaging a trauma informed approach to data collection it was largely possible to overcome longstanding concerns about addressing survivor needs and re-traumatisation. In doing so, their work embraces ideals of epistemic justice offering a nuanced insight to theory and politics of knowing through engagement with a historically excluded group. In their second paper, Barker et al. draw on efforts to create conditions to provide an affirming environment for survivors by delivering trauma informed training to non-specialist employees at the IICSA. Participants felt such organisational considerations facilitated safety and trusting relations with survivors, and the authors theorised elements of testimonial sensibility were secured through this therapeutic culture. Alyce et al. echo the significance of testimonial sensibility within a survivor approach to participatory research. They offer a nuanced and reflexive insight about the way this approach avoids hermeneutical barriers of misunderstanding and misinterpretation, which in turn provides the foundation for testimonial justice. It is an approach which imbued safety, minimised mistrust, and helped to remove the pain of epistemic silence.

This Research Topic has explored different forms of epistemic injustices and how epistemic justice can be advanced in mental health theory, practice, or research from different disciplinary perspectives. However, as various contributors make clear, advancing epistemic justice is a work in progress and needs to centre lived experience and seek to involve those who have been marginalised. A major limitation of this Research Topic is the absence of papers from low- and middle-income countries. We hope that this Research Topic is further developed with contributions from voices of experience in these countries.

## Author contributions

KN: Writing – original draft, Writing – review & editing. AS: Writing – original draft, Writing – review & editing. U-KS: Writing – original draft, Writing – review & editing. CK: Writing – review & editing.

## Footnotes

1 Beresford, P., 2003. *It's our lives: A short theory of knowledge, distance and experience*. London: OSP for Citizen Press. Available at: It’s Our Lives: A short theory of knowledge, distance and experience - Shaping Our Lives.

2 Fricker, M., 2007. *Epistemic injustice: Power and the ethics of knowing*. Oxford University Press.

3 Allen, A., 2017. Power/knowledge/resistance: Foucault and epistemic injustice. In *The Routledge handbook of epistemic injustice* (pp. 187-194). Routledge.

4 Crenshaw, K.W., 2017. *On intersectionality: Essential writings*. The New Press.

